# Liver Abscess Caused by Infection with Community-Acquired *Klebsiella quasipneumoniae* subsp. *quasipneumoniae*

**DOI:** 10.3201/eid2203.151466

**Published:** 2016-03

**Authors:** Sebastien Breurec, Benedicte Melot, Bruno Hoen, Virginie Passet, Kinda Schepers, Sylvaine Bastian, Sylvain Brisse

**Affiliations:** Institut Pasteur de la Guadeloupe, Les Abymes, France (S. Breurec);; Centre Hospitalier Universitaire de Pointe-à-Pitre/les Abymes, Pointe-à-Pitre, France (S. Breurec, B. Melot, B. Hoen, K. Schepers, S. Bastian);; Université des Antilles, Faculté de Médecine Hyacinthe Bastaraud, Pointe-à-Pitre (S. Breurec, B. Hoen);; Inserm CIC 14-24, Pointe à Pitre (B. Melot, B. Hoen, K. Schepers);; Institut Pasteur, Paris, France (V. Passet, S. Brisse);; Centre National de la Recherche Scientifique, Paris (V. Passet, S. Brisse)

**Keywords:** Klebsiella quasipneumoniae subsp. quasipneumoniae, liver abscess, integrative and conjugative element ICEKp1, capsular serotype K1, bacteria

## Abstract

We report a case of pyogenic liver abscess caused by community-acquired *Klebsiella quasipneumoniae* subsp. *quasipneumoniae*. The infecting isolate had 2 prominent features of hypervirulent *K. pneumoniae* strains: the capsular polysaccharide synthesis region for K1 serotype and the integrative and conjugative element ICE*Kp1,* which encodes the virulence factors yersiniabactin, salmochelin, and RmpA.

The syndrome of pyogenic liver abscess caused by community-acquired *Klebsiella pneumoniae* (CA-KLA) infection has been described mainly in Asia, particularly in Taiwan. Infection is caused by hypervirulent strains of particular clonal groups (CG); prominent among the clonal groups is CG23 of capsular serotype K1 (*1*,*2*). Although intestinal colonization is probably a prerequisite for disease, the gate of entry leading to infection and mechanism by which it occurs are unknown (*3*). A novel species of the genus *Klebsiella* closely related to *K. pneumoniae*, *K. quasipneumoniae*, was recently described (*4*); the species is divided into 2 subspecies, but its pathogenicity is not well known. Until now, *K. quasipneumoniae* has only been isolated from persons with hospital-acquired infections or carriage (*4*–*6*). We report a case of liver abscess caused by community-acquired *K. quasipneumoniae* subsp. *quasipneumoniae*.

On June 21, 2014, a 65-year-old man was admitted to the medical center of Basse-Terre, Guadeloupe (French West Indies), with a history of fever, vomiting, and joint pain. He also had a history of coronary heart disease, type 2 diabetes, and essential hypertension. The patient had not previously been hospitalized in 2014. He was given analgesic drugs and was discharged. Five days later, he again visited the medical center with persistent fever. Clinical examination showed a painful, red left eye; congestive heart failure; and a tender, enlarged spleen. Laboratory analysis showed elevated biological values for serum C-reactive protein (328 mg/L), serum procalcitonin (18 mg/L), leukocytes (21.5 cells/μL), polymorphonuclear leukocytes (20.5 cells/μL), platelets (30 cells/μL), aspartate aminotransferase (8 × the upper limit of normal [U/L]), alanine aminotransferase (3.5 × U/N), total bilirubin (43 μmol/L), and serum creatinine (170 μmol/L. Urine and blood cultures were negative, and findings of chest radiograph and abdominal ultrasound were unremarkable. Treatment was begun with intravenous amoxicillin/clavulanate. 

On June 28, the patient was transferred to the university medical center at Pointe-à-Pitre, Guadeloupe. Ophthalmic examination revealed uveitis in the left eye. The diagnoses of leptospirosis with ocular involvement and bacterial sepsis were considered, and the antimicrobial agent was changed to ceftriaxone daily. On July 4, the diagnosis of leptospirosis was regarded as most likely, and antimicrobial drug therapy was narrowed to amoxicillin. However, on July 8, the eye condition (endophthalmitis and orbital cellulitis) worsened, and the antimicrobial drugs were switched to ceftazidime and levofloxacin. 

On July 17, the patient’s general condition had improved, although endophthalmitis persisted. All microbiological samples remained negative, as did all test results for *Leptospira* spp. Drug treatment was stopped. On July 28, because the patient reported recurring/constant abdominal pain in the right upper quadrant of the abdomen, a computed tomography scan was performed; it showed a 35 × 35 × 60 mm abscess in liver segments 5 and 6. The abscess was drained on July 30, yielding pus that, when cultured, grew *K. pneumoniae* (API 20NE system strip; bioMérieux, Marcy-l’Etoile, France). The patient responded well and was treated as an outpatient with oral moxifloxacin (400 mg/d) for an additional 2 weeks. He recovered, albeit with permanent monocular blindness.

To determine the genotypic characteristics of the *Klebsiella* isolate (SB4935), we obtained a genomic sequence using a 2 × 300 nt paired-end protocol on an MiSeq instrument (Illumina, San Diego, CA. USA). Reads were assembled using a CLCbio assembler (Aarhus, Denmark) into 66 contigs of an average coverage depth of 47 of high-quality nucleotides. The draft genome sequence was 5.2 Mb in length and 57% rich in guanine-cytosine content. The genomic sequence was submitted to the European Nucleotide Archive (accession no. PRJEB9601). Multilocus sequence typing (MLST) (*7*), core genome MLST, antimicrobial drug resistance, and virulence genes were searched by using the BIGSdb *Klebsiella* genome database (http://bigsdb.web.pasteur.fr/klebsiella) (*8*). Capsular typing was performed by slide agglutination. Susceptibility to antimicrobial drugs was determined by disk diffusion. The genome was annotated by using the RAST server (*9*). Comparison with genome of NTUH-K2044 (*10*) was performed with the Artemis Comparison Tool (http://www.sanger.ac.uk/software/artemis/ACT).

Phylogenetic analysis of the 7-gene MLST sequences showed that isolate SB4935 belongs to *K. quasipneumoniae* subsp. *quasipneumoniae* (sequence type 446) (*4*). Notably, the strain possessed a capsular polysaccharide synthesis (*cps*) region, typical of strains of capsular serotype K1 (*3*). Comparison with the *cps* region of *K. pneumoniae* K1 reference strain NTUH-K2044 showed complete conservation of genes across the entire *cps* cluster (from genes *galF* to *uge*, with 92% to 100% protein identity, depending on the gene). Strain SB4935 reacted against anti-K1 serum. Thus, horizontal transfer of the entire K1 *cps* region had occurred, either between *K. pneumoniae* and *K. quasipneumoniae* or from another unidentified lineage. 

Furthermore, the SB4935 genome comprised a 76-kb DNA genomic island that displayed features typical of a horizontally acquired region: 1) chromosomal insertion into the asn-tRNA locus, 2) an integrase gene, and 3) flanking 16-bp direct repeats. This genomic island was highly similar (89%–100% protein identity) to the integrative and conjugative element (ICE) ICE*Kp1* of *K. pneumoniae* NTUH-K2044 (*11*) and coded for the following virulence factors: a yersiniabactin iron-uptake system, the regulator of mucoid phenotype RmpA, and salmochelin (*iroBCDN* cluster). In addition, genes for the conjugative transfer of the island were present. The insertion was found at the same location in NTUH-K2044 and SB4935 genomes; that is, immediately downstream of a tRNA-Asn locus adjacent to gene KP1_3578 coding for a sodium:proton antiporter. These results indicate horizontal gene transfer of the ICE*Kp1* at the same location in both strains.

Strain SB4935 harbored other typical virulence factors of *K. pneumoniae*. The *iutA* gene, which codes for the ferric aerobactin receptor, was present, but not *iucABCD,* which is involved in aerobactin biosynthesis. This finding suggests that isolate SB4935 can benefit from the production of aerobactin by neighboring strains (*12*). In addition, the genome harbored clusters *mrkABCDFHIJ* for type III fimbriae, involved in adhesion and biofilm formation, and *fimABCDEFGHI,* coding for type 1 fimbriae involved in urinary tract adhesion (*3*). No resistance gene was detected in the SB4935 genome other than *bla*_OKP_, the β-lactamase gene of *K. quasipneumoniae* (*13*). This finding was consistent with the antimicrobial drug susceptibility profile (resistance only to ampicillin, ticarcillin, and piperacillin).

The clinical features of this case were similar to those of other published cases of CA-KLA (*14*). The pathogen causing endophthalmitis was not cultured, however. Although uncommon, endogenous endophthalmitis, which occurs by hematogenous dissemination, has been reported as a complication of hypervirulent *K. pneumoniae* liver abscess (*2*,*3*). In addition, because the patient did not receive antimicrobial drugs when blood cultures were obtained, the cultures’ negative results might be due to low-level bacteremia. 

The isolate we identified had several prominent features of hypervirulent *K. pneumoniae* strains, including the *cps* cluster for K1 capsule synthesis and ICE*Kp1*-encoding yersiniabactin, salmochelin, and RmpA. Serotype K1 is the most frequent capsular type of *K. pneumoniae* associated with CA-KLA (*1*,*3*). ICE*Kp1* has been more prevalent in strains associated with CA-KLA than in non–tissue invasive strains (*11*). Yersiniabactin is one of the most prominent features associated with invasive *K. pneumoniae* strains (*6*), and animal models support its strong pathologic contribution (*15*). Thus, horizontal transfer of high pathogenicity features into multidrug-resistant *K. pneumoniae* strains is concerning (*3*,*8*). Conjugative transfer of ICE*Kp1* from NTUH-K2044 to *Escherichia coli* and *K. pneumoniae* has been demonstrated (*11*). 

Although we could not establish the history of transfer events, we identified high-virulence features in a close phylogenetic neighbor of *K. pneumoniae*. Further work is needed to clarify reservoirs of high pathogenicity elements and the mechanisms of transfer that contribute to the emergence of highly virulent *Klebsiella* strains.
